# Cooperative Binding

**DOI:** 10.1371/journal.pcbi.1003106

**Published:** 2013-06-27

**Authors:** Melanie I. Stefan, Nicolas Le Novère

**Affiliations:** 1 Division of Biology, California Institute of Technology, Pasadena, California, United States of America; 2 Babraham Institute, Babraham, Cambridge, United Kingdom; University of Toronto, Canada

## Abstract

Molecular binding is an interaction between molecules that results in a stable association between those molecules. **Cooperative binding** occurs if the number of binding sites of a macromolecule that are occupied by a specific type of ligand is a nonlinear function of this ligand's concentration. This can be due, for instance, to an affinity for the ligand that depends on the amount of ligand bound. Cooperativity can be positive (supralinear) or negative (infralinear). Cooperative binding is most often observed in proteins, but nucleic acids can also exhibit cooperative binding, for instance of transcription factors. Cooperative binding has been shown to be the mechanism underlying a large range of biochemical and physiological processes.

This is a “Topic Page” article for *PLOS Computational Biology*.

## History and Mathematical Formalisms

### Christian Bohr and the Concept of Cooperative Binding

**Figure 1:**
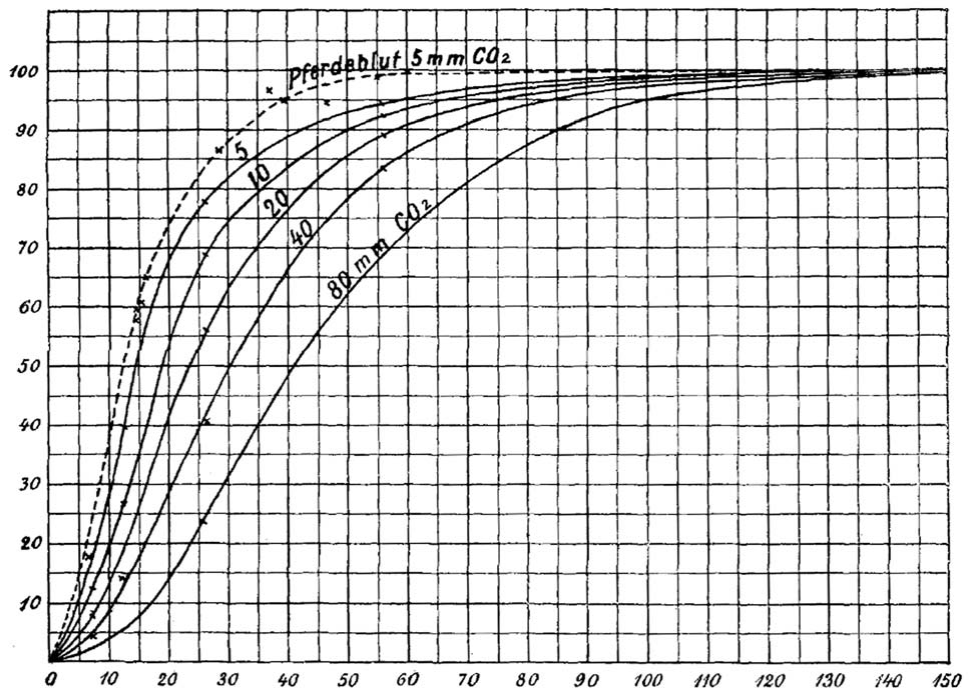
Original figure from Christian Bohr [1], showing the sigmoidal increase of oxyhemoglobin as a function of the partial pressure of oxygen.

In 1904, Christian Bohr studied hemoglobin binding to oxygen under different conditions [1]. When plotting hemoglobin saturation with oxygen as a function of the partial pressure of oxygen, he obtained a sigmoidal (or “S-shaped”) curve, see Figure 1. This indicates that the more oxygen is bound to hemoglobin, the easier it is for more oxygen to bind—until all binding sites are saturated. In addition, Bohr noticed that increasing CO2 pressure shifted this curve to the right—i.e., higher concentrations of CO_2_ make it more difficult for hemoglobin to bind oxygen [1]. This latter phenomenon, together with the observation that hemoglobin's affinity for oxygen increases with increasing pH, is known as the Bohr effect.

A receptor molecule is said to exhibit cooperative binding if its binding to ligand scales nonlinearly with ligand concentration. Cooperativity can be positive (if binding of a ligand molecule increases the receptor's apparent affinity, and hence increases the chance of another ligand molecule binding) or negative (if binding of a ligand molecule decreases affinity and hence makes binding of other ligand molecules less likely). Figure 1 is a chart of the “fractional occupancy” 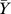 of a receptor with a given ligand, which is defined as the quantity of ligand-bound binding sites divided by the total quantity of ligand binding sites:
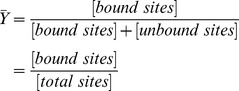



If 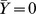, then the protein is completely unbound, and if 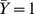, it is completely saturated. If the plot of 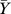 at equilibrium as a function of ligand concentration is sigmoidal in shape, as observed by Bohr for hemoglobin, this indicates positive cooperativity. If it is not, no statement can be made about cooperativity from looking at this plot alone.

The concept of cooperative binding only applies to molecules or complexes with more than one ligand binding site. If several ligand binding sites exist, but ligand binding to any one site does not affect the others, the receptor is said to be noncooperative. Cooperativity can be homotropic, if a ligand influences the binding of ligands of the same kind, or heterotropic, if it influences binding of other kinds of ligands. In the case of hemoglobin, Bohr observed homotropic positive cooperativity (binding of oxygen facilitates binding of more oxygen) and heterotropic negative cooperativity (binding of CO_2_ reduces hemoglobin's facility to bind oxygen).

Throughout the twentieth century, various frameworks have been developed to describe the binding of a ligand to a protein with more than one binding site and the cooperative effects observed in this context (reviewed by Wyman, J. and Gill, 1990 [2]).

### The Hill Equation

**Figure 2:**
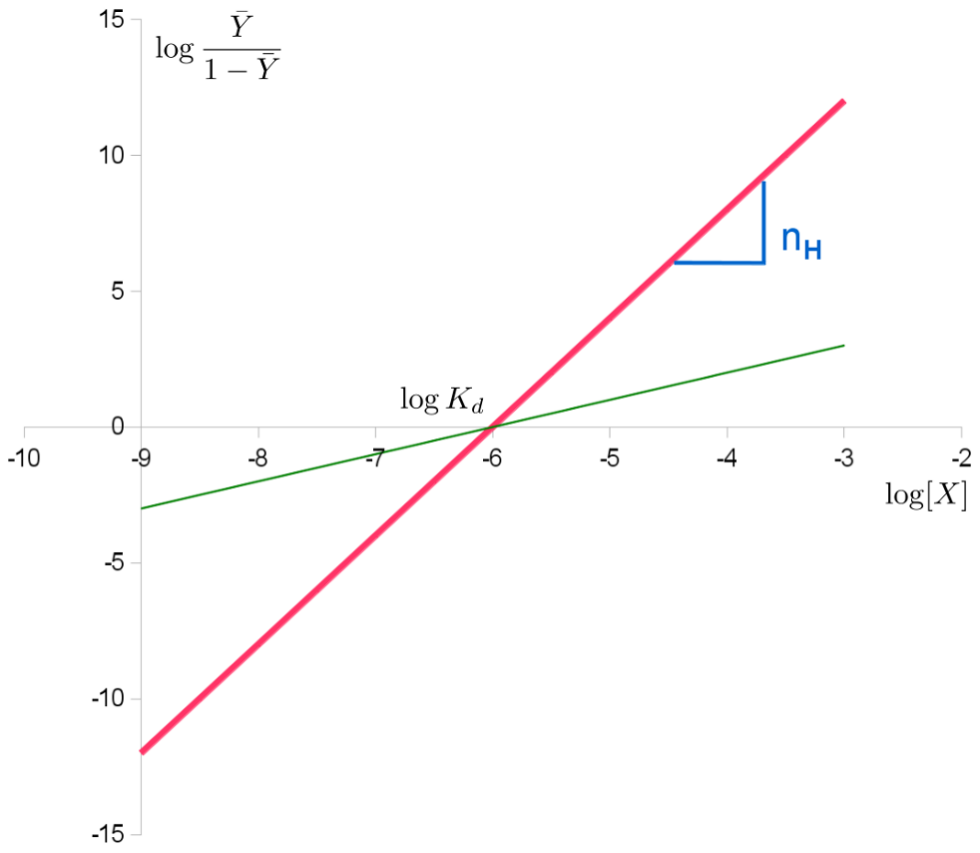
Hill plot of the Hill equation in red, showing the slope of the curve being the Hill coefficient and the intercept with the x-axis providing the apparent dissociation constant. The green line shows the noncooperative curve.

The first description of cooperative binding to a multisite protein was developed by A.V. Hill [3]. Drawing on observations of oxygen binding to hemoglobin and the idea that cooperativity arose from the aggregation of hemoglobin molecules, each one binding one oxygen molecule, Hill suggested a phenomenological equation that has since been named after him

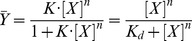
where *n* is the “Hill coefficient,” [*X*] denotes ligand concentration, *K* denotes an apparent association constant (used in the original form of the equation), and *K_d_* is an apparent dissociation constant (used in modern forms of the equation). If *n*<1, the system exhibits negative cooperativity, whereas cooperativity is positive if *n*>1. The total number of ligand binding sites is an upper bound for *n*. The Hill equation can be linearized as:
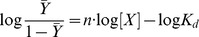



The “Hill plot” is obtained by plotting 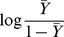 versus log[*X*]. In the case of the Hill equation, it is a line with slope *n_H_* and intercept *log*(*K_d_*) (see Figure 2). This means that cooperativity is assumed to be fixed, i.e., it does not change with saturation. It also means that binding sites always exhibit the same affinity, and cooperativity does not arise from an affinity increasing with ligand concentration.

### The Adair Equation

G. S. Adair found that the Hill plot for hemoglobin was not a straight line, and hypothesized that cooperativity was not a fixed term, but dependent on ligand saturation [4]. Having demonstrated that hemoglobin contained four hemes (and therefore binding sites for oxygen), he worked from the assumption that fully saturated hemoglobin is formed in stages, with intermediate forms with one, two, or three bound oxygen molecules. The formation of each intermediate stage from unbound hemoglobin can be described using an apparent macroscopic association constant *K_i_*. The resulting fractional occupancy can be expressed as:
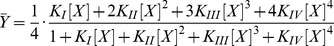



Or, for any protein with *n* ligand binding sites:
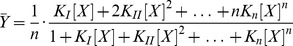
where *n* denotes the number of binding sites and each *K_i_* is a combined association constant, describing the binding of *i* ligand molecules.

### The Klotz Equation

Working on calcium binding proteins, Irving Klotz deconvoluted Adair's association constants by considering stepwise formation of the intermediate stages, and tried to express the cooperative binding in terms of elementary processes governed by mass action law [5], [6]. In his framework, *K_1_* is the association constant governing binding of the first ligand molecule, *K_2_* the association constant governing binding of the second ligand molecule (once the first is already bound), etc. For 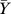, this gives:
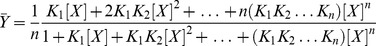



It is worth noting that the constants *K_1_*, *K_2_*, and so forth do not relate to individual binding sites. They describe *how many* binding sites are occupied, rather than *which ones*. This form has the advantage that cooperativity is easily recognised when considering the association constants. If all ligand binding sites are identical with a microscopic association constant *K*, one would expect 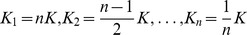 (that is 
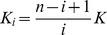
) in the absence of cooperativity. We have positive cooperativity if *K_i_* lies above these expected values for *i*>1.

The Klotz equation (which is sometimes also called the Adair-Klotz equation) is still often used in the experimental literature to describe measurements of ligand binding in terms of sequential apparent binding constants [5].

### Pauling Equation

By the middle of the twentieth century, there was an increased interest in models that would not only describe binding curves phenomenologically, but offer an underlying biochemical mechanism. Linus Pauling reinterpreted the equation provided by Adair, assuming that his constants were the combination of the binding constant for the ligand (*K* in the equation below) and energy coming from the interaction between subunits of the cooperative protein (*α* below) [7]. Pauling actually derived several equations, depending on the degree of interaction between subunits. Based on wrong assumptions about the localisation of hemes, he opted for the wrong one to describe oxygen binding by hemoglobin, assuming the subunits were arranged in a square. The equation below provides the equation for a tetrahedral structure, which would be more accurate in the case of hemoglobin:
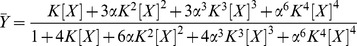



### The KNF Model

Based on results showing that the structure of cooperative proteins changed upon binding to their ligand, Daniel Koshland. and colleagues [8] refined the biochemical explanation of the mechanism described by Pauling [7]. The Koshland-Némethy-Filmer (KNF) model assumes that each subunit can exist in one of two conformations: active or inactive. Ligand binding to one subunit would induce an immediate conformational change of that subunit from the inactive to the active conformation, a mechanism described as “induced fit” [9]. Cooperativity, according to the KNF model, would arise from interactions between the subunits, the strength of which varies depending on the relative conformations of the subunits involved. For a tetrahedric structure (they also considered linear and square structures), they proposed the following formula:
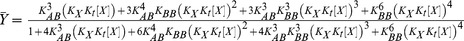
where *K_X_* is the constant of association for *X*, *K_t_* is the ratio of *B* and *A* states in the absence of ligand (“transition”), and *K_AB_* and *K_BB_* are the relative stabilities of pairs of neighbouring subunits relative to a pair where both subunits are in the *A* state (note that the KNF paper actually presents *N_s_*, the number of occupied sites, which is here 4 times 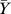).

### The MWC Model

**Figure 3:**
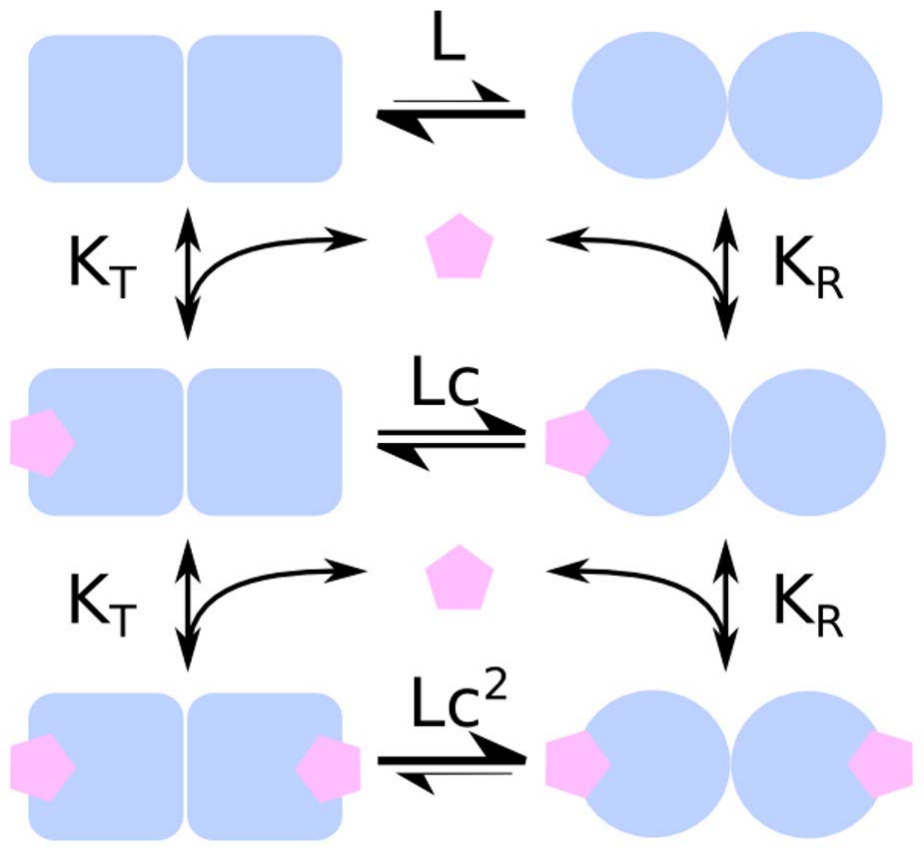
Reaction scheme of a Monod-Wyman-Changeux model of a protein made up of two protomers. The protomer can exist under two states, each with a different affinity for the ligand. *L* is the ratio of states in the absence of ligand, *c* is the ratio of affinities.

**Figure 4:**
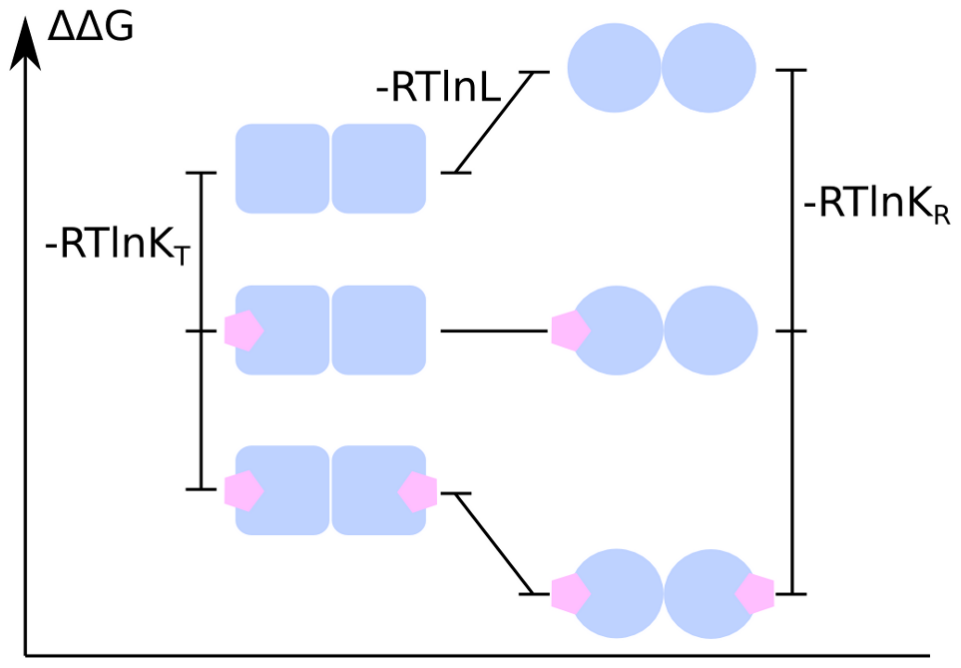
Energy diagram of a Monod-Wyman-Changeux model of a protein made up of two protomers. The larger affinity of the ligand for the *R* state means that the latter is preferentially stabilised by the binding.

The Monod-Wyman-Changeux (MWC) model for concerted allosteric transitions [10] went a step further by exploring cooperativity based on thermodynamics and three-dimensional conformations. It was originally formulated for oligomeric proteins with symmetrically arranged, identical subunits, each of which has one ligand binding site. According to this framework, two (or more) interconvertible conformational states of an allosteric protein coexist in a thermal equilibrium. The states—often termed tense (*T*) and relaxed (*R*)—differ in affinity for the ligand molecule. The ratio between the two states is regulated by the binding of ligand molecules that stabilises the higher-affinity state. Importantly, all subunits of a molecule change states at the same time, a phenomenon known as “concerted transition.” The MWC model is illustrated in Figure 3 and Figure 4.

**Figure 5:**
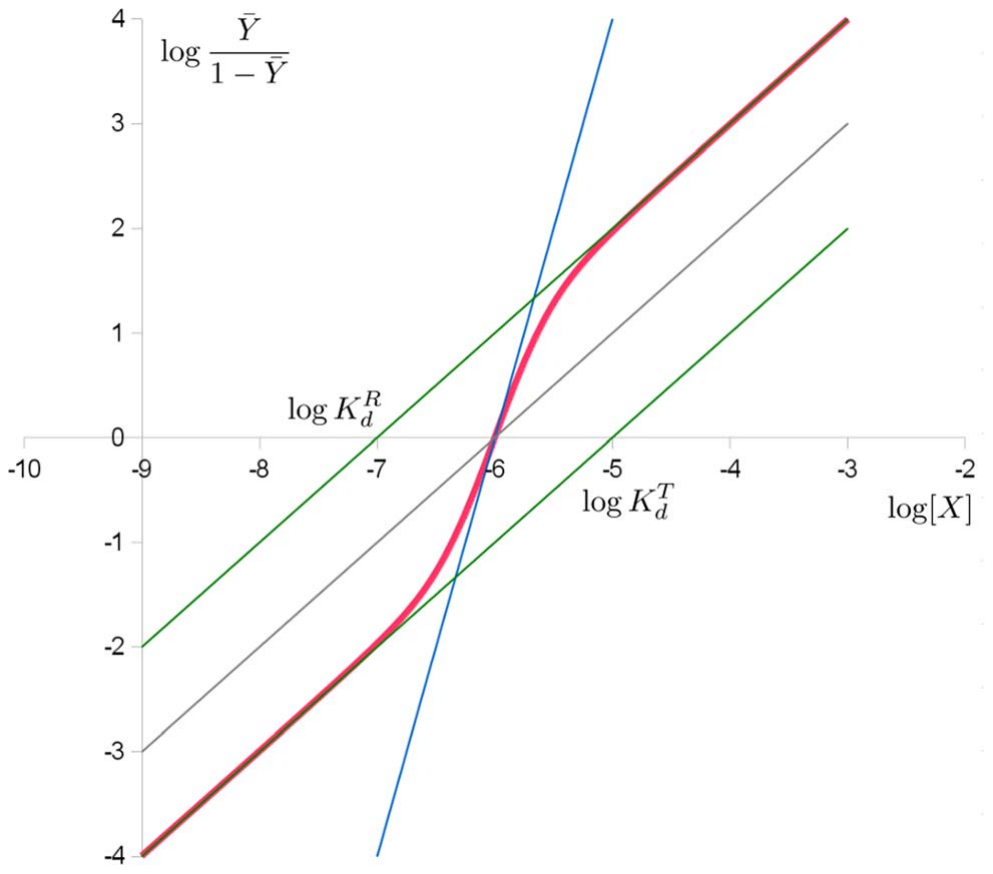
Hill plot of the MWC binding function in red, of the pure *T* and *R* state in green. As the conformation shifts from *T* to *R*, so does the binding function. The intercepts with the x-axis provide the apparent dissociation constant as well as the microscopic dissociation constants of the *R* and *T* states.

The allosteric isomerisation constant *L* describes the equilibrium between both states when no ligand molecule is bound: 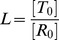. If *L* is very large, most of the protein exists in the *T* state in the absence of ligand. If *L* is small (close to one), the *R* state is nearly as populated as the *T* state. The ratio of dissociation constants for the ligand from the *T* and *R* states is described by the constant *c*: 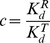. If *c* = 1, both *R* and *T* states have the same affinity for the ligand and the ligand does not affect isomerisation. The value of *c* also indicates how much the equilibrium between *T* and *R* states changes upon ligand binding: the smaller *c*, the more the equilibrium shifts towards the *R* state after one binding. With 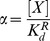, fractional occupancy is described as:
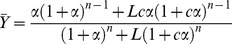



The sigmoid Hill plot of allosteric proteins (shown in Figure 5) can then be analysed as a progressive transition from the *T* state (low affinity) to the *R* state (high affinity) as the saturation increases. The Hill coefficient also depends on saturation, with a maximum value at the inflexion point. The intercepts between the two asymptotes and the y-axis allow us to determine the affinities of both states for the ligand.

In proteins, conformational change is often associated with activity, or activity towards specific targets. Such activity is often what is physiologically relevant or what is experimentally measured. The degree of conformational change is described by the state function 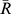, which denotes the fraction of protein present in the *R* state. As the energy diagram illustrates, 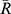 increases as more ligand molecules bind. The expression for 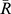 is:
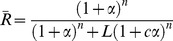



A crucial aspect of the MWC model is that the curves for 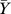 and 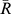 do not coincide [11], i.e., fractional saturation is not a direct indicator of conformational state (and hence, of activity). Moreover, the extents of the cooperativity of binding and the cooperativity of activation can be very different: an extreme case is provided by the bacteria flagella motor with a Hill coefficient of 1.7 for the binding and 10.3 for the activation [12], [13]. The supralinearity of the response is sometimes called ultrasensitivity.

If an allosteric protein binds to a target that also has a higher affinity for the *R* state, then target binding further stabilises the *R* state, hence increasing ligand affinity. If, on the other hand, a target preferentially binds to the *T* state, then target binding will have a negative effect on ligand affinity. Such targets are called allosteric modulators.

Since its inception, the MWC framework has been extended and generalised. Variations have been proposed, for example, to cater for proteins with more than two states [14], proteins that bind to several types of ligands [15], [16] or several types of allosteric modulators [16], and proteins with nonidentical subunits or ligand-binding sites [17].

## Examples of Cooperative Binding

The list of molecular assemblies that exhibit cooperative binding of ligands is very large, but some examples are particularly notable for their historical interest, their unusual properties, or their physiological importance.

**Figure 6:**
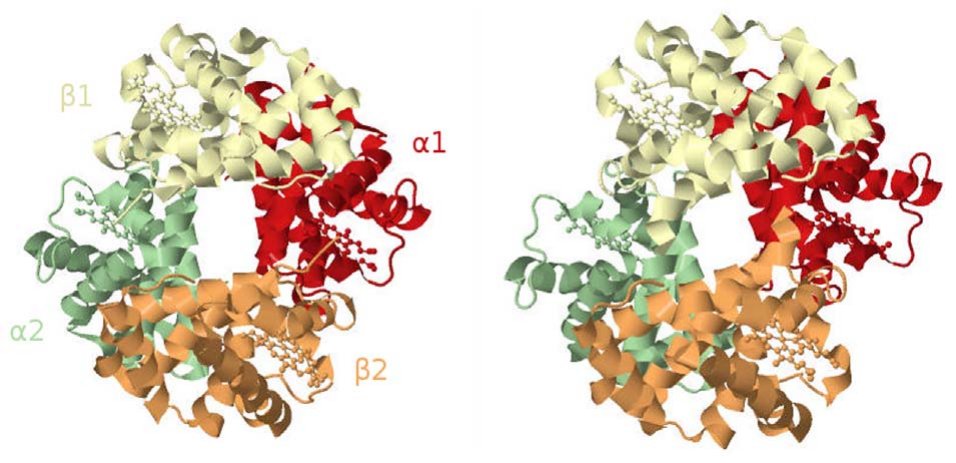
Cartoon representation of the protein hemoglobin in its two conformations: “tensed (*T*)” on the left corresponding to the deoxy form (derived from Protein Data Bank entry 1LFL) and “relaxed (*R*)” on the right corresponding to the oxy form (derived from Protein Data Bank entry 1LFT). Alpha globins are red and green, while beta globins are yellow and orange.

As described in the historical section, the most famous example of cooperative binding is hemoglobin. Its quaternary structure, solved by Max Perutz using X-ray diffraction [18], exhibits a pseudo-symmetrical tetrahedron carrying four binding sites (hemes) for oxygen (see Figure 6). Many other molecular assemblies exhibiting cooperative binding have been studied in great detail.

### Multimeric Enzymes

The activity of many enzymes is regulated by allosteric effectors. Some of these enzymes are multimeric and carry several binding sites for the regulators.

Threonine deaminase was one of the first enzymes suggested to behave like hemoglobin [19] and shown to bind ligands cooperatively [20]. It was later shown to be a tetrameric protein [21].

Another enzyme that was suggested early to bind ligands cooperatively is aspartate trans-carbamylase [22]. Although initial models were consistent with four binding sites [23], its structure was later shown to be hexameric by William Lipscomb and colleagues [24].

### Ion Channels

Most ion channels are formed by several identical or pseudo-identical monomers or domains, arranged symmetrically in biological membranes. Several classes of such channels whose opening is regulated by ligands exhibit cooperative binding of these ligands.

It was suggested as early as 1967 [25] (when the exact nature of those channels was still unknown) that the nicotinic acetylcholine receptors bound acetylcholine in a cooperative manner due to the existence of several binding sites. The purification of the receptor [26] and its characterization demonstrated a pentameric structure with binding sites located at the interfaces between subunits, confirmed by the structure of the receptor binding domain [27].

Inositol triphosphate (IP3) receptors form another class of ligand-gated ion channels exhibiting cooperative binding [28]. The structure of those receptors shows four IP3 binding sites symmetrically arranged [29].

### Multisite Molecules

**Figure 7:**
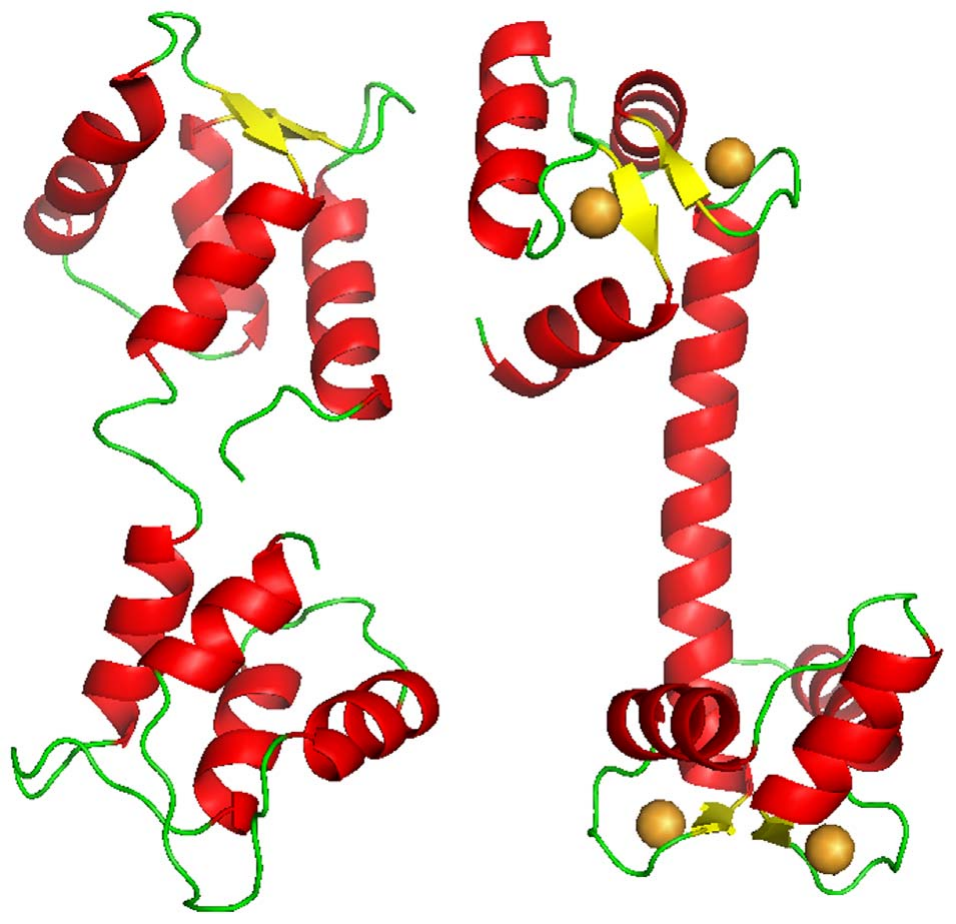
Cartoon representation of the protein calmodulin in its two conformations: “closed” on the left (derived from PDB id: 1CFD) and “open” on the right (derived from PDB id: 3CLN). The open conformation is represented bound with 4 calcium ions (orange spheres).

Although most proteins showing cooperative binding are multimeric complexes of homologous subunits, some proteins carry several binding sites for the same ligand on the same polypeptide. One such example is calmodulin (Figure 7). One molecule of calmodulin binds four calcium ions cooperatively [30]. Its structure presents four EF-hand domains [31], each one binding one calcium ion. Interestingly, the molecule does not display a square or tetrahedron structure, but is formed of two lobes, each carrying two EF-hand domains.

### Transcription Factors

Cooperative binding of proteins onto nucleic acids has also been shown. A classical example is the binding of the lambda phage repressor to its operators, which occurs cooperatively [32], [33]. Other examples of transcription factors exhibit positive cooperativity when binding their target, such as the repressor of the TtgABC pumps [34] (n = 1.6).

Conversely, examples of negative cooperativity for the binding of transcription factors were also documented, as for the homodimeric repressor of the Pseudomonas putida cytochrome P450cam hydroxylase operon (n = 0.56) [35].

### Conformational Spread and Binding Cooperativity

Early on, it was argued that some proteins, especially those consisting of many subunits, could be regulated by a generalised MWC mechanism, in which the transition between *R* and *T* state is not necessarily synchronized across the entire protein [36]. In 1969, Wyman [37] proposed such a model with “mixed conformations” (i.e., some protomers in the *R* state, some in the *T* state) for respiratory proteins in invertebrates.

Following a similar idea, the conformational spread model by Duke and colleagues [38] subsumes both the KNF and the MWC models as special cases. In this model, a subunit does not automatically change conformation upon ligand binding (as in the KNF model), nor do all subunits in a complex change conformations together (as in the MWC model). Conformational changes are stochastic with the likelihood of a subunit switching states depending on whether or not it is ligand bound and on the conformational state of neighbouring subunits. Thus, conformational states can “spread” around the entire complex.

## Supporting Information

Text S1 Version history of the text file.
(XML)

Text S2 Peer reviews and response to reviews. Human-readable versions of the reviews and authors' responses are available as comments on this article.
(XML)
